# Mechanisms of Lifespan Extension by Sirt6 in Drosophila melanogaster

**DOI:** 10.1093/geroni/igaa057.2617

**Published:** 2020-12-16

**Authors:** Jackson Taylor, Jason Wood, Matthew Finn, Julianna Liu, Evan Mizerak, Sarah Gordon, Corinne Hutfilz, Stephen Helfand

**Affiliations:** Brown University, Providence, Rhode Island, United States

## Abstract

Sirt6 is a multifunctional enzyme that regulates numerous cellular processes connected to longevity. Overexpressing Sirt6 extends lifespan in mice, but the underlying cellular mechanisms are unclear. Here, we used the powerful genetic tools and short lifespan of Drosophila melanogaster to better understand the precise mechanisms by which Sirt6 regulates longevity. Sirt6 OE in flies produces robust extension of median lifespan in both sexes. Molecular and biochemical analyses reveal that Sirt6 OE reduces expression of genes involved in protein synthesis, including many Myc target genes, via epigenetic regulation. We will further discuss our findings on the connection between Sirt6, Myc, and the molecular regulation of protein synthesis and lifespan, as well as additional Sirt6 longevity mechanisms we identified, including autophagy and silencing of transposable elements.

